# Complete genome sequence data for *Klebsiella pneumoniae* superbug strain KAB03 isolated from ready-to-eat vegetable salads in Central Uganda

**DOI:** 10.1016/j.dib.2026.113094

**Published:** 2026-07-22

**Authors:** Abubaker Kawooya, Chinyere Nkemjika Anyanwu, Emmanuel Eilu, Hussein Kafeero, Adam Ahmed Adam, Mariam Oyedeji Amusa, Danladi Makeri, Martin Odoki, Rasheed Omotayo Adeyemo, Michael Ben Okon, Ismail Abiola Adebayo, Saheed Adekunle Akinola

**Affiliations:** a Department of Microbiology/Immunology, Kampala International University Western Campus, Kampala, Uganda; b Clarke International University, Kampala, Uganda; c Department of Mechatronics and Biomedical Engineering, Faculty of Engineering, Kyambogo University, P.O. Box 1, Kyambogo, Uganda; d Kampala International University, Box 20000, Ggaba Road, Kasanga, Kampala, Uganda; e Department of Applied Sciences, School of Sciences, Nkumba University, Entebbe, Uganda; f Department of Microbiology and Immunology, School of Medicine, Health & Life Sciences, King Ceasor University, Kampala, Uganda; g Department of Microbiology and Parasitology, School of Medicine and Pharmacy, College of Medicine and Health Sciences, University of Rwanda, Butare, Rwanda; h Department of Biochemistry, Faculty of Biomedical Sciences, Kampala International University, Ishaka, Uganda; i Department of Medical Biochemistry, Molecular Biology, and Genetics, School of Medicine and Pharmacy, College of Medicine and Health Sciences, University of Rwanda, Butare, Rwanda

**Keywords:** One health, Infection control warning, Antimicrobial resistance, High-throughput sequencing, Superbugs, Market-to-mouth foods

## Abstract

We present whole-genome sequence data for the *Klebsiella pneumoniae* strain KAB03, isolated from Ready-To-Eat Vegetable Salads in Kampala District, Central Uganda. Genomic DNA was extracted and sequenced using the Illumina platform. The assembled genome is 5,772,016 base pairs long with a GC content of 56.7%. The data set includes annotated genes related to cross-resistance to conventional antibiotics and widely used disinfectants or antiseptics, such as *bla*_SHV-1_, *KpnF, FosA6, OmpK37, MdtQ, oqxA*, and *KpnG* / *KpnH*. The genome sequence and accompanying metadata are available at https://www.ncbi.nlm.nih.gov/nuccore/JBWXCM000000000. This data set is suitable in defining a high-risk multidrug-resistance (MDR), public health surveillance, and comparative genomics among *K. pneumonia* isolates from the environment.

Specifications Table

**Table:** 

Subject	Health Sciences, Medical Sciences & Pharmacology
Specific subject area	Bacterial genomics; Food safety; Antimicrobial resistance.
Type of data	Genome assembly metrics, annotated gene data, Raw Sequence Reads (FASTQ), Assembled (FASTA), Annotated (GenBank format)
Data collection	Ready-To-Eat Vegetable Salad samples were collected from roadside sellers. About 25 g of the food sample was enriched in 225 mL buffered peptone water (BPW; Oxoid, Hampshire, U.K.) for 24 h at 37 °C. Following enrichment, a loopful of broth was streaked onto MacConkey agar and incubated at 37 °C for 24 h under aerobic conditions. DNA was extracted using the QIAamp DNA Mini Kit and quantified using the Qubit 4 Fluorometer (Thermo Fisher) and the NanoDrop ND-1000. Sequencing was performed on the Illumina NovaSeq 6000 platform (2 × 150 bp). Reads were quality-checked with FastQC, trimmed with Trimmomatic, and assembled using SPAde.
Data source location	Ready-to-Eat vegetable salads were collected from Roadside sellers in Kampala City (0°18′34“N 32°34′40″ E), Uganda.
Data accessibility	Data is publicly available at the NCBI repository: BioProject accession: PRJNA1435052 BioSample accession: SAMN56432132 Genome accession: JBWXCM000000000 Direct URLs to data: https://www.ncbi.nlm.nih.gov/nuccore/JBWXCM000000000
Related research article	None

## 1 Value of the Data

• This dataset provides the complete genome of a *Klebsiella pneumoniae* strain isolated from Ready-To-Eat Vegetable Salad in a low-resource African setting, contributing to the limited genomic data available for such an emerging foodborne pathogen in sub-Saharan Africa.
• Researchers can use this data to identify genetic elements associated with resistance to access and watch group antibiotics in food isolates.
• The data support the One Health approach by linking food safety with clinical resistance patterns.
• This genome sequence offers a practical reference for bioinformatics pedagogical exercises, algorithmic testing, and the comparative benchmarking of annotation pipelines, especially when applied to datasets characterizing polyclonal or polymicrobial infections in low-resource settings.

## 2 Background

The increasing consumption of ready-to-eat (RTE) vegetable salads is a significant public health concern because these foods undergo minimal processing, which often fails to eliminate opportunistic pathogens [1]. Among these, multidrug-resistant (MDR) *Klebsiella pneumoniae* has emerged as a critical food-chain contaminant [2,3]. While traditionally recognized as a nosocomial pathogen, *K. pneumoniae* is increasingly isolated from RTE salads, serving as a reservoir for antimicrobial resistance (AMR) genes and virulence factors that can be transmitted to humans via the fecal-oral route [4,5]. In food safety systems, the presence of *K. pneumoniae* in RTE vegetable salads poses a dual threat: it can serve as a vehicle for clinical infections and as a medium for horizontal gene transfer of resistance determinants within the gut microbiome. Understanding the genomic architecture of foodborne strains is essential for tracking transmission dynamics and identifying emerging hypervirulent lineages.

This data article presents the Whole Genome Sequencing (WGS) of a *K. pneumonia* KAB03-strain isolated from RTE vegetable salads. High-throughput sequencing provides a high-resolution view of the strain's sequence type, capsular serotypes (K and O types), and its entire resistome. By making this genomic data available, we provide a reference for comparative genomic analyses, enabling researchers to evaluate evolutionary relationships among environmental, foodborne, and clinical isolates. This data is foundational for developing targeted biocontrol strategies, including phage-based interventions, to enhance the safety of the RTE vegetable salad supply chain.

## 3 Data Description

The final assembled genome of *K. pneumoniae* KAB03 is **5,772,016** **bp** long with a GC content of 56.7**%**. It contains 5882 predicted protein-coding genes, **78** tRNA genes, 3 complete 23S rRNA gene, L50 contigs (7), and N50 contigs (324,195 bp). Quality analysis of genome using CheckM (v1.2.3) showed **100%** completeness and 0.9% contamination. Fast Whole-Genome Similarity (ANI) Estimation showed that the closest type strain is *K. pneumoniae* (GCA_000240185.2) with value >98% and genome coverage of 99.25%, confirming the close relatedness and species-level identity. Screening with the Comprehensive Antibiotic Resistance Database (CARD) revealed several resistance genes as listed in Table 1. The presence of important genes such as *marA, KpnE*, and *leuO*, which are associated with cross-resistance to conventional antibiotics and widely used disinfectants or antiseptics raises significant food safety concerns, as it implies these bacteria can withstand routine sanitization procedures commonly used in commercial food preparation. The presence of these genetic markers also suggests that the bacteria are highly adapted to survive in challenging environments, enabling them to persist on food contact surfaces and within biofilms on processing equipment. Because traditional chemical disinfectants may be ineffective against such robust strains. As such, there is an urgent need to explore alternative biocontrol methods such as bacteriophages.

**Table 1:** Resistance analysis of *K. pneumonia* KAB03 using Comprehensive Antibiotic Resistance Database (CARD).

Gene	Drug class(es)	Resistance mechanism	Key features
*bla*_SHV-1_	Cephalosporins, penicillins, beta-lactams	Antibiotic inactivation	**Perfect hit** (100% identity), classic beta-lactamase
*KpnF*	Macrolides, aminoglycosides, cephalosporins, tetracyclines, peptides, rifamycins, disinfectants	Efflux pump (SMR)	**Perfect hit** (100% identity), very broad substrate range
*FosA6*	Fosfomycin (phosphonic acid)	Antibiotic inactivation	**Strict hit** (98.56% identity), clinically relevant for fosfomycin resistance
*OmpK37*	Monobactams, carbapenems, cephalosporins, penicillins, beta-lactams	Reduced permeability to antibiotic	**Strict hit** (99.47% identity), porin mutation-mediated resistance
*MdtQ*	Monobactams, carbapenems, cephalosporins, penicillins, beta-lactams	Reduced permeability to antibiotic	**Perfect hit** (100% identity), outer membrane porin
*oqxA*	Fluoroquinolones, glycylcyclines, tetracyclines, diaminopyrimidines, nitrofurans	Efflux pump (RND)	**Strict hit** (99.49% identity), plasmid-mediated resistance
*KpnG / KpnH*	Macrolides, fluoroquinolones, aminoglycosides, carbapenems, cephalosporins, penicillins, peptides	Efflux pump (MFS)	**Strict hits** (99.74% and 93.95% identity), broad multidrug efflux

## 4 Experimental Design, Materials and Methods

### 4.1 Sample collection and isolation

Strain KAB03 was isolated from a ready-to-eat (RTE) vegetable salad collected from Roadside sellers in Kampala (0°18′34“N 32°34′40″ E), Central Uganda. The RTE vegetable salad comprises shredded cabbage, carrots, cucumber, and tomatoes, and is prepared without the addition of cheese or dressing. A 25 g sample of the salad was aseptically homogenized in 225 mL of sterile Buffered Peptone Water (Oxoid, UK) using a stomacher for 2 min and incubated aerobically under static condition at 37 °C for 24 h. A loopful of the enriched culture was streaked onto MacConkey agar (Microxpress®, India) and incubated overnight at 37 °C under the same conditions [6]. Pure colonies that appeared pink and mucoid on the MacConkey agar were presumptively identified as *Klebsiella pneumoniae*. These isolates were further subjected to biochemical tests, including indole, citrate, and triple sugar iron (TSI) agar tests [[7], [8], [9]].

### 4.2 DNA extraction and whole-genome sequencing

Overnight culture of a single colony of the confirmed *K. pneumoniae* isolate was performed in Luria Bertani broth (Himedia, India) at 37 °C with agitation at 200 rpm. Following incubation, bacterial cells were collected by centrifugation at 12,000 × *g* for 10 min. Genomic DNA was extracted using the Wizard® Genomic DNA kit (Promega) following the manufacturer's protocol [10]. Library preparation was conducted using the Illumina DNA Prep kit. DNA integrity was assessed via electrophoresis on a 1% (w/v) agarose gel. DNA concentration was quantified using the Qubit dsDNA High Sensitivity Assay Kit (Thermo Fisher Scientific), and the purity was evaluated with a NanoDrop ND-1000 spectrophotometer (Thermo Fisher Scientific) [11]. Sequencing was performed on the Illumina NovaSeq 6000 platform with 2 × 150 bp paired-end reads. Library preparation was conducted using the Nextera XT kit (Illumina, USA).

### 4.3 Genome assembly and annotation

A total of **5,772,016** paired-end reads were obtained after sequencing. All reads passed quality assessment using FastQC (v0.12.1) [12,13]. Trimmomatic (v0.39) [14] was used to remove adapter sequences and low-quality bases. All high-quality trimmed reads were used for de novo assembly with SPAdes (v3.15.3) [15], resulting in 98 contigs with N50 contig of 324,195 bp. The genome was annotated using the NCBI Prokaryotic Genome Annotation Pipeline (PGAP) [16].

## Limitations

Not Applicable.

## Ethics Statement

This study did not involve human subjects or animal experiments. However, institutional approval for food safety research was obtained from the Kampala International University Research Ethics Committee (Reference number: KIU-2023-272) and the Uganda National Council for Science and Technology (Reference Number: HS3986ES).

## CRediT Author Statement

**Abubaker Kawooya** : Conceptualization, Methodology, Supervision, Investigation, Formal Analysis, Writing Original Draft; **Jesca Nakavuma** : Conceptualization, Methodology, Supervision, Review & Editing; **Chinyere Anyanwu:** Software, Data Curation, Visualization; **Emmanuel Eilu:** Resources, Validation, Software, Formal Analysis, Review & Editing; **Hussein Kafeero** : Resources, Validation, Software, Formal Analysis, Review & Editing; **Adam Ahmed Adam** : Resources, Validation, Software, Formal Analysis; **Mariam Oyedeji Amusa**: Resources, Validation, Software, Formal Analysis, **Danladi Makeri**: Resources, Validation, Software, Formal Analysis, Review & Editing; **Martin Odoki**: Laboratory Work, Data Collection, Review & Editing; **Simon Peter Kaweesa** : Laboratory Work, Data Collection, Review & Editing; **Rasheed Omotayo Adeyemo**: Data Curation, Software, Visualization, Review & Editing; **Michael Ben Okon**: Data Curation, Software, Visualization, Review & Editing; **Ismail Abiola Adebayo**: Laboratory Work, Data Collection, Review & Editing; **Saheed Adekunle Akinola:** Conceptualization, Methodology, Supervision, Project Administration. All authors have read and approved the final version of the manuscript.

## Data Availability

NCBIKlebsiella pneumoniae strain KAB03, whole genome shotgun sequencing project (Original data).
